# A melting, encapsulated, calcified pulmonary nodule in a healthy 51-year-old woman: a case report

**DOI:** 10.1186/s12880-020-00448-5

**Published:** 2020-05-01

**Authors:** Hsu-Chao Chang, Yi-Hsin Lee, Mei-Chen Yang

**Affiliations:** 1 Department of Radiology, Taipei Tzu Chi Hospital, Buddhist Tzu Chi Medical Foundation, New Taipei, Taiwan; 2 Department of Anatomy Pathology, Taipei Tzu Chi Hospital, Buddhist Tzu Chi Medical Foundation, New Taipei, Taiwan; 3 School of Medicine, Tzu Chi University, Hualien, Taiwan; 4 Division of Pulmonary Medicine, Department of Internal Medicine, Taipei Tzu Chi Hospital, Buddhist Tzu Chi Medical Foundation, New Taipei, Taiwan

**Keywords:** Distal airway, Foreign body, Imaging findings, Case report

## Abstract

**Background:**

Foreign body aspiration is less common in healthy adults, which makes diagnosis difficult. Early detection of smaller/sharp foreign bodies in the distal airway is more difficult because patients might have no symptoms and imaging studies could appear normal.

Here we describe the course of a small, sharp foreign body (chicken bone) lodged in the distal airway of a healthy middle-aged woman. The chicken bone was initially thought to be an old calcified tuberculoma. However, it was encased in a dilated bronchus without obvious surrounding lymphadenitis or parenchymal infiltration, and it melted with time. Two years later, histopathological examination revealed that the calcified lesion was an aspirated chicken bone with a concomitant tuberculoma.

**Case presentation:**

A 51-year-old woman showed an old calcified tuberculoma in the upper right lung lobe during routine examinations. It was “encased” in a dilated bronchus, although it was not raised from the surrounding lung parenchyma. The size of the calcified part decreased (“melted”) with time, and the surrounding inflammation progressed 2 years later, a phenomenon never described in association with tuberculosis. Bronchoscopy revealed a fragment of chicken bone lodged in the next two branches of the upper right posterior bronchus. Surgical segmentectomy was performed, and histopathological examination revealed that the calcified lesion was formed by a fragment of chicken bone as well as a tuberculoma. Eventually, the patient recalled an episode of choking on a chicken bone 5 years ago; she believed that she had coughed it out completely at that time.

**Conclusions:**

The “melting” and “encased” phenomena observed in the present case could be useful imaging findings for early detection of small foreign body aspiration in the distal airway.

## Background

Non-asphyxiating foreign body aspiration is less common in healthy adults. The diagnosis is difficult because symptoms are often absent and nonspecific and chest radiography (CXR) may appear normal because of the organic composition of the foreign bodies [1–3]. Chest computed tomography (CT) is more sensitive than CXR, but with limited application in the identification of small and distal lesions [4–6]. Flexible bronchoscopy is useful for accessing foreign bodies in the proximal airway, although it cannot access foreign bodies in the distal airway, especially if they are too small or dislodged too distally to be visualized and removed [7]. Foreign bodies in the distal airway are almost impossible to be identified early and are often found incidentally during histopathological examination after surgery for other reasons [2, 5, 8].

Here we describe the course of a small, sharp foreign body lodged in the distal airway of a healthy middle-aged woman who denied any previous history of respiratory symptoms and choking. The calcified lesion was initially thought to be an old calcified tuberculoma. However, the calcified nodule was encased in a dilated bronchus and not raised from the lung parenchyma, and the calcified part reduced in size while the surrounding inflammation progressed with time; these phenomena were unusual for tuberculosis. Histopathological examination of surgical segmentectomy specimens revealed that the calcified lesion was formed by both a tuberculoma and a foreign body.

## Case presentation

**Fig. 1:**
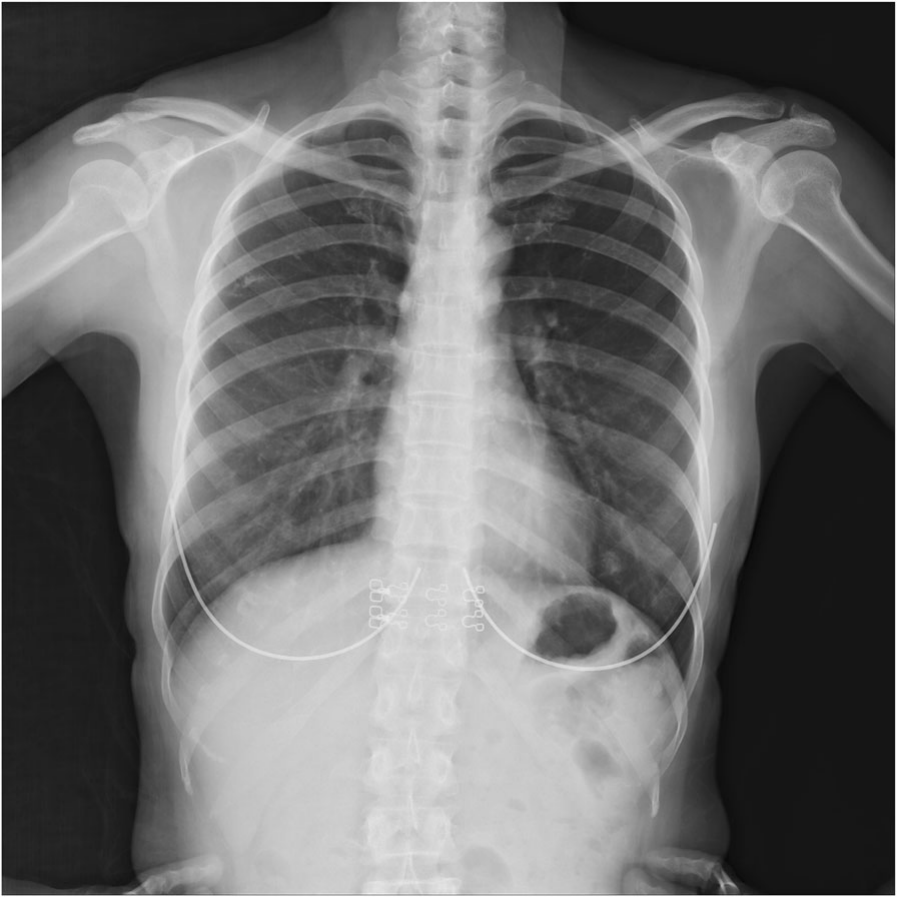
Initial chest radiograph. The initial chest radiograph shows a calcified nodule in the upper right pulmonary lobe (yellow arrowhead)

**Fig. 2:**
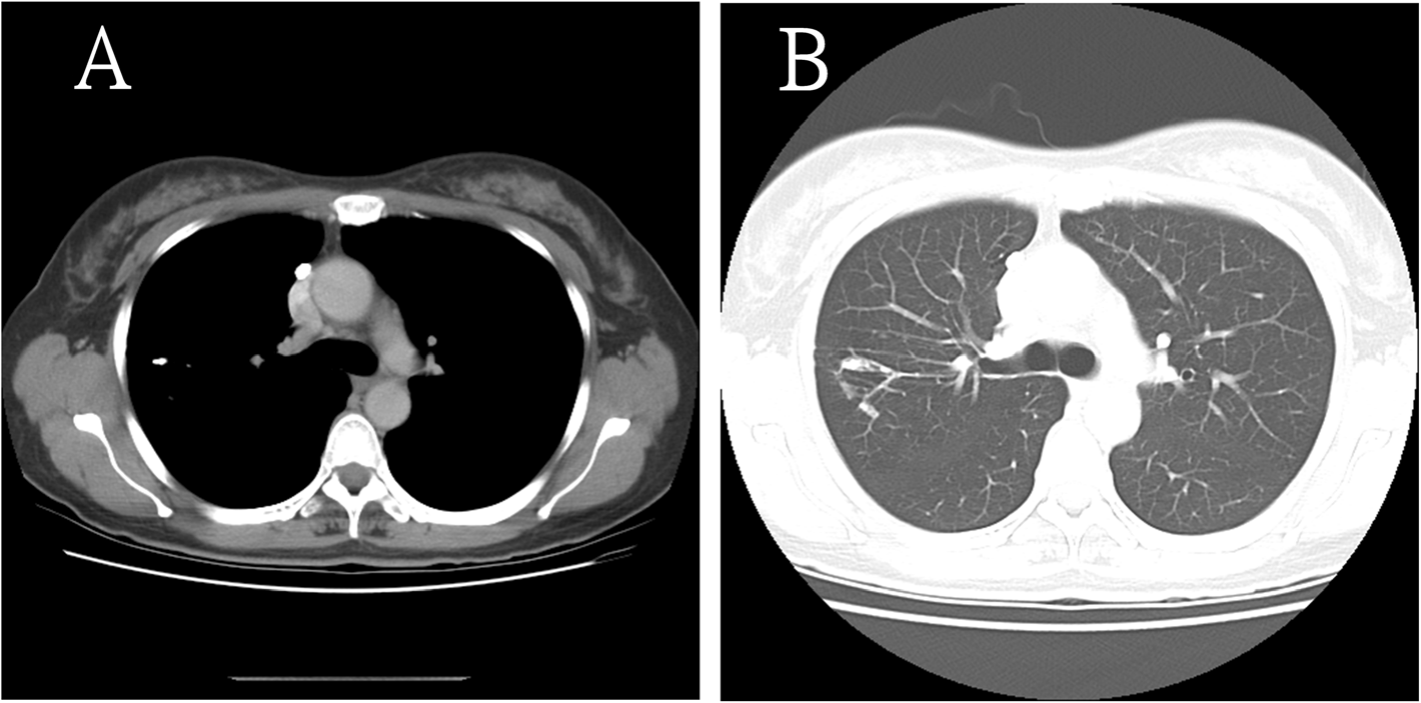
Initial chest computed tomography. Initial chest computed tomography shows a calcified nodule in the upper right lobe in the mediastinal window (yellow arrowhead) (**a**). The nodule is encased within the dilated bronchus (yellow arrowheads), with parenchymal infiltration in the lung window (blue arrowheads) (**b**)

A healthy 51-year-old woman underwent routine CXR and chest CT examinations, which revealed an old calcified pulmonary nodule in the right upper lobe (Fig. 1). On chest CT, the lesion measured 7.1 × 3.4 mm (Fig. 2a, b). She was informed that it was merely an old calcified tuberculoma and no further diagnostic procedures were needed because she did not have any discomfort. We also advised her to visit for a follow-up 1 year later.

**Fig. 3:**
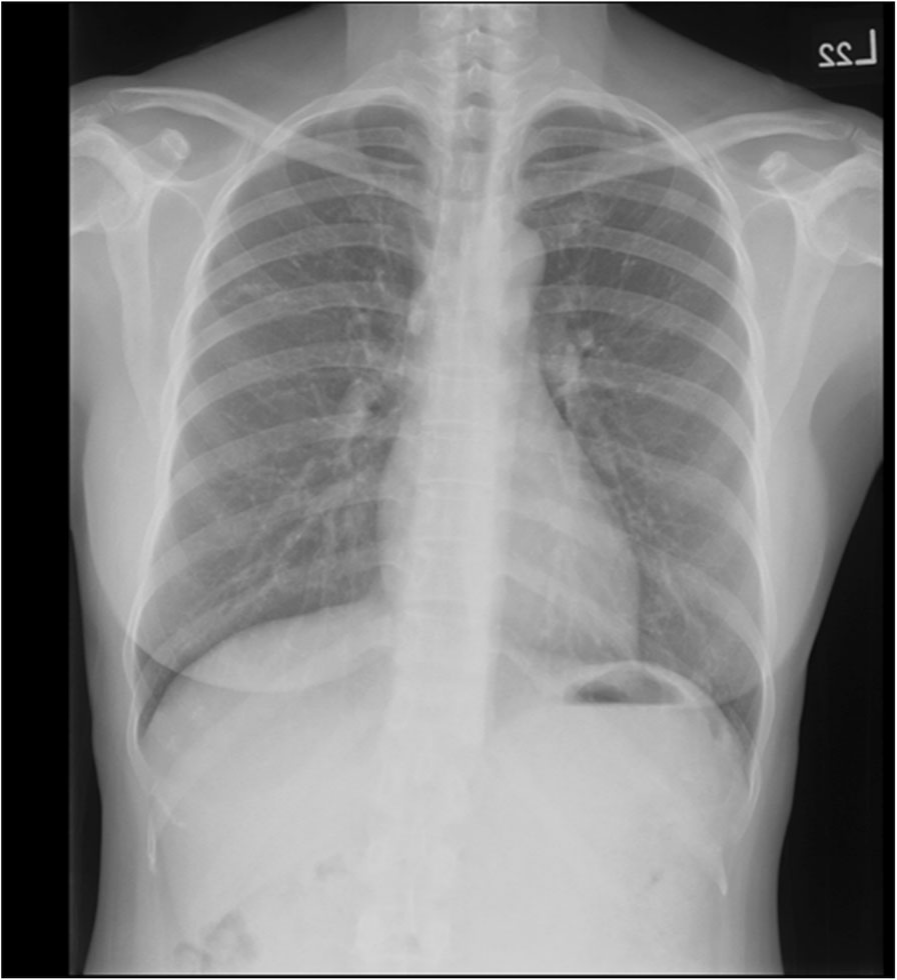
Follow-up chest radiograph obtained 2 years later. Two years later, a chest radiograph showed a change in the shape and decrease in the size of the calcified nodule in the upper right lobe (yellow arrowhead)

**Fig. 4:**
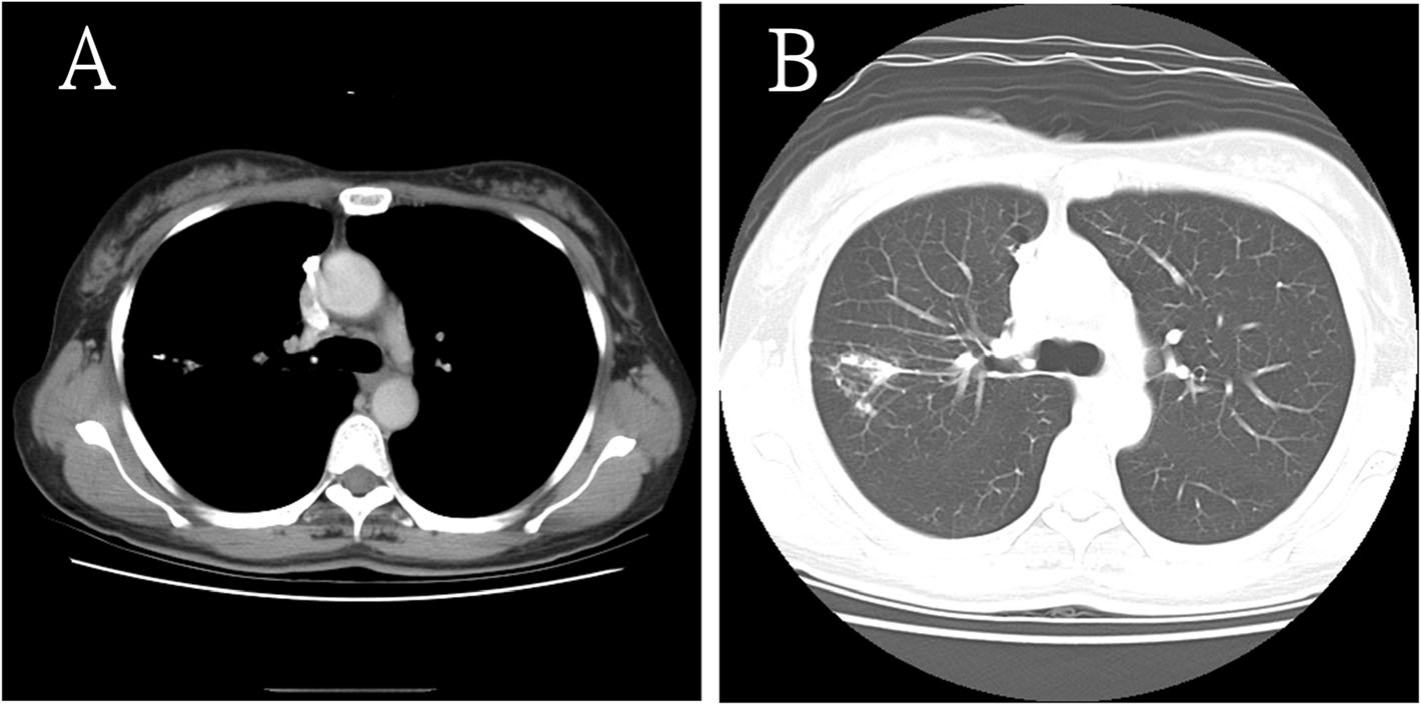
Follow-up chest computed tomography performed 2 years later. Two year later, chest computed tomography shows that the calcified nodule has “melted” over time and remains encased in the dilated bronchus (yellow arrowhead) (**a**). It is accompanied by aggregating parenchymal infiltration (blue arrowheads) (**b**)

**Fig. 5:**
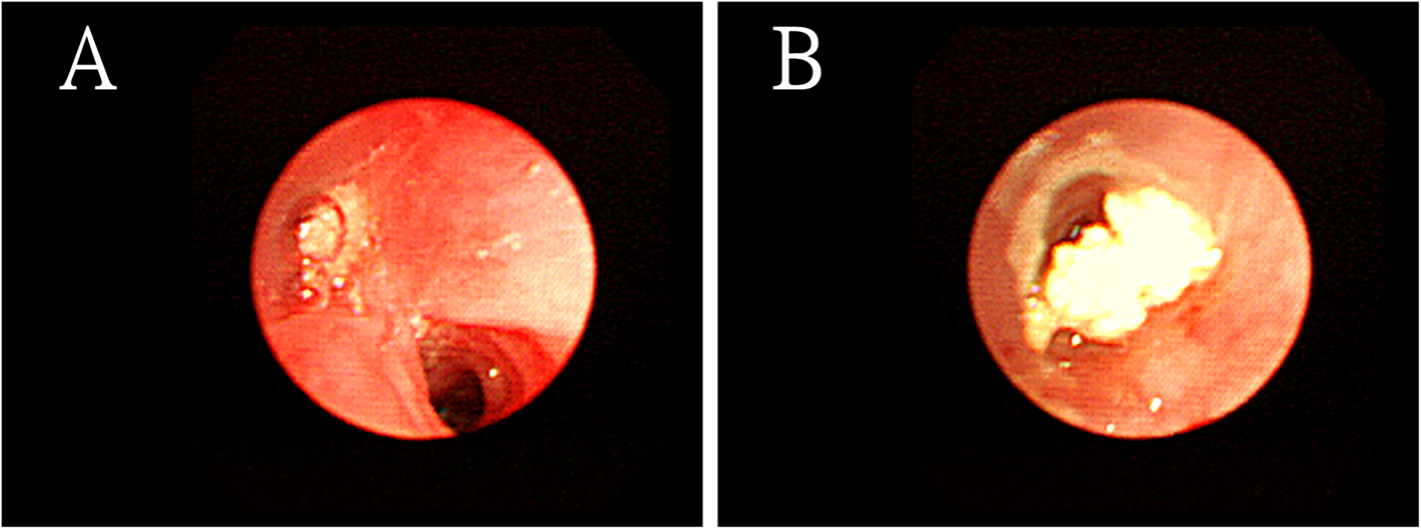
Bronchoscopy imaging. Adult bronchoscopy reveals obscure pus in the bronchus of the upper right posterior segment (**a**). A pediatric bronchoscope is advanced further after pus removal, and it shows a whitish, irregularly shaped lesion lodged firmly in the next two branches of the upper right posterior segment (**b**)

**Fig. 6:**
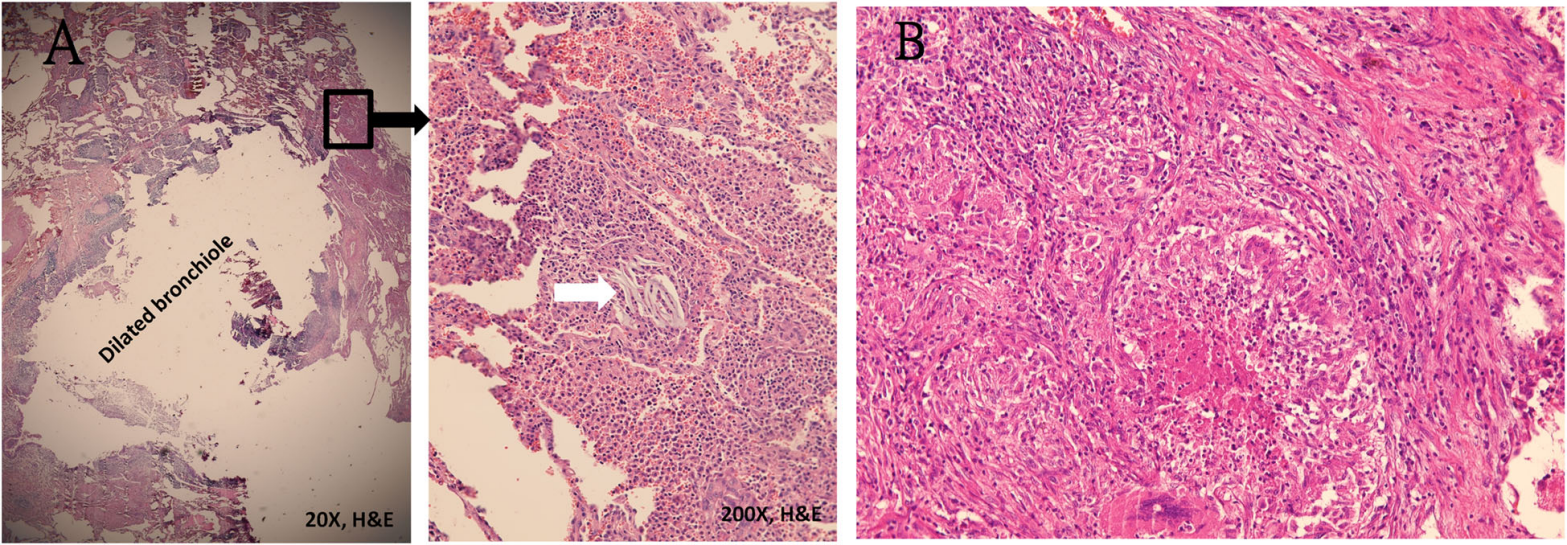
Histopathological examination. Histological examination revealed marked lymphplasma infiltration in the mucosa of the dilated bronchiole, mucin and inflammatory exudates in the lumen (**a**, left, hematoxylin and eosin stain, 20X). A few small foreign body-like material and aggregation of the foamy histiocytes were noted in the peripheral parenchyma of the dilated bronchiole, which may be the residual fragments of the chicken bone (**a**, right, white arrowhead, hematoxylin and eosin stain, 200X). In another specimen a bit far away of the dilated bronchiole, a tuberculoma was found with abundant caseous necrosis surrounded by granulomatous inflammation and multinuclear Langhan’s giant cells (**b**, hematoxylin and eosin stain, 200X)

However, she returned 2 years later, and follow-up CXR showed a change in the size and shape of the calcified nodule (Fig. 3). Chest CT revealed that the size of the calcified part decreased to 5.6 × 2.3 mm. In other words, it had “melted” over time. Moreover, it was surrounded by aggregating pulmonary inflammation (Fig. 4a, b). The calcified nodule was also found to be “encased” in a dilated bronchus, although it was not raised from the surrounding lung parenchyma. Flexible fiberoptic bronchoscopy was performed with an adult bronchoscope, which revealed some obscure pus obstructing the bronchus of the upper right posterior segment (Fig. 5a). The adult bronchoscope could not be advanced further because of the difficult angle of the upper right posterior segment. Thus, we used a pediatric flexible fiberoptic bronchoscope to remove the discharge and found a whitish, sharp-edged lesion lodged tightly in the next two branches of the upper right posterior segment (Fig. 5b). We failed to extract it using pediatric biopsy forceps. Segmentectomy was performed. Histological examination revealed marked lymphplasma infiltration in the mucosa of the dilated bronchiole, mucin and inflammatory exudates in the lumen (Fig. 6a, left). A few small foreign body-like material and aggregation of the foamy histiocytes were noted in the peripheral parenchyma of the dilated bronchiole, which may be the residual fragments of the chicken bone (Fig. 6a, right). In another specimen a bit far away of the dilated bronchiole, a tuberculoma was found with abundant caseous necrosis surrounded by granulomatous inflammation and multinuclear Langhan’s giant cells (Fig. 6b). An old tuberculoma with a concurrent decaying chicken bone in the distal airway was the final diagnosis. Because of the progressive changes in the calcified component of the lesion on chest CT, we believed that the aspirated chicken bone, and not the tuberculoma itself, was the origin of the calcified lesion. Eventually, the patient reluctantly recalled a previous episode of chicken bone aspiration 5 years ago. She was discharged 5 days after surgery and prescribed anti-tuberculosis drugs for 6 months. She remained healthy and showed no problems during the annual follow-up visits in the following 10 years.

## Discussion and conclusion

Foreign body aspiration is less common in healthy adults, which makes diagnosis difficult, especially in the absence of an obvious choking episode or typical symptoms [1, 2]. The patient in the present case was healthy and did not have a risk of choking or specific symptoms. Initial CXR and chest CT revealed a calcified lesion, which was (reasonably) thought to be an old tuberculoma. However, the calcified lesion “melted” with time and was encased in a dilated bronchus. A calcified tuberculoma would presumably be located in lung parenchyma and would not have undergone decalcification with time. Initially, the patient denied a history of choking, but later recalled an episode of chicken bone aspiration about 3 years ago, after pathological examination confirmed the diagnosis. It escaped her attention since she remained asymptomatic for 3 years and believed that she had already coughed it out completely. The chicken bone must have been irregular and sharp initially, which allowed it to get dislodged into the orifice of upper right posterior bronchus, where it did not cause airway obstruction. It was located in the center of the bronchus of the upper right posterior pulmonary lobe. The “encased” phenomenon shown by the long calcified nodule hints at the possibility of foreign body aspiration, because old calcified granulomas are supposed to be located in the lung parenchyma and are not “encased” within the bronchus. While the chicken bone decayed with time, the residual fragments were dislodged more distally, causing distal airway obstruction. The natural course of undetected distal airway foreign body had not been reported. We think that the presence of the “melting” and “encased” phenomena on chest imaging may be useful for the early diagnosis of foreign body aspiration.

Organic substances, particularly chicken or fish bones, are the most common types of foreign bodies that are aspirated into the lower airway [1, 4]. However, this could vary, depending on the dietary habits in different countries; vast differences have been found between Chinese adults those in western countries [9, 10].

A foreign body wedged distally in the distal airway can be innocuous [4], and a choking history is important for early identification. However, only 25–38% of adults with foreign bodies in the lower airway remember a choking event [11, 12]. Our patient initially denied a choking episode because she was confident that she had coughed it out completely. Therefore, she did not pay attention and almost forgot about this episode.

Flexible bronchoscopy is the definitive method for the diagnosis and removal of foreign bodies in the airway [4]. However, a foreign body is often surrounded by friable granulation tissue, which should be evaluated carefully before attempting [4]. Although we could see the foreign body with the pediatric bronchoscope, we were still unable to view the whole relationship between the bronchus and foreign body completely. It might have been dangerous to extract the chicken bone at that time. Moreover, we would have the diagnosis of pulmonary tuberculosis even if we removed the chicken bone successfully. A previous study reported an incidental intra-operative diagnosis of a retained foreign body (plastic whistle), which was initially misdiagnosed as pulmonary tuberculosis [2]. We also misdiagnosed the calcified lesion as pulmonary tuberculosis initially. Interestingly, the patient also had pulmonary tuberculosis.

Nor Hisyam had reported a case with a sharp fish bone dislodged on to the right vocal cord because it was T-shaped with two sharp pointed ends. The T-shaped fish bone seemed to be larger than our patient’s chicken bone [13]. Sharp foreign bodies, such as fish or chicken bones, commonly lodge in the tonsil, base of tongue, vallecula or pyriform fossa and rarely get dislodged into the laryngopharynx and lower airway. However, a sharp foreign body that is small enough, would have to undergo alterations in size, to get dislodged into the distal airway.

Pulmonary calcification can be divided into two major categories: metastatic and dystrophic calcifications and pulmonary alveolar microlithiasis, a rare disorder [14]. Metastatic pulmonary calcification is defined by calcium salt deposition in normal lung tissues and commonly found in end-stage kidney disease requiring hemodialysis [15]. Dystrophic pulmonary calcification originates from previously injured lung tissue, including infectious or non-infectious granulomatous diseases, and accounts for the majority of pulmonary calcifications [16]. *Mycobacterium tuberculosis* infections often generate granulomatous tissue resulting in intrathoracic dystrophic calcifications in the form of parenchymal granulomas, mediastinal lymph node calcification, and calcified fibronodular areas in the lung [16, 17]. In our patient, the calcified lesion was encased in a dilated bronchus and not raised from the lung parenchyma; therefore, it could not be a tuberculosis-related parenchymal granuloma. Moreover, there was no fibronodular change in the lung parenchyma surrounding the dilated bronchus, and it could come from the calcified fibronodular areas of lung. Broncholithiasis, defined as a calcified lesion within the bronchial lumen, is often caused by erosion due to the extrusion of calcified peribronchial lymph nodes, and it could be considered as a differential diagnosis for our patient [18]. However, there were no enlarged lymph nodes surrounding the dilated bronchus, and the bronchial mucosa was relatively smooth and intact on bronchoscopy images; this decreased the possibility of broncholithiasis.

Granuloma originates from any infectious process and has the potential to become calcified over time, because calcium has a tendency to collect in injured tissue [19]. Therefore, a tuberculoma could undergo classification. Foreign body-related non-caseating granulomatous inflammation could be another type of granuloma and can show calcification with time, as calcium deposits in the inflammatory tissue for a long period. However, the histopathological findings in our patient did not reveal any calcium deposits within the foreign body-related non-caseating granulomatous inflammation. The residual fragment of the chicken bone was completely located in the central part of the non-caseating granulomatous inflammation. Therefore, we believe that the calcified part of the nodule originated from the chicken bone, not from the tuberculoma.

This case was interesting and educational because the calcified pulmonary lesion was initially thought to be an old calcified tuberculoma and was later found to be a chicken bone on bronchoscopy. Eventually, both conditions were identified on histopathological examination. The “melting” calcified lesion on the imaging was finally thought to be a manifestation of the decaying chicken bone, not the calcified granuloma. The presence of “melting” and “encased” phenomena on chest CT might be useful imaging findings that can aid in the early detection of foreign body aspiration.

## Data Availability

All data and figures generated in this study are included in this published article.

## Abbreviations

CT: Computed tomography
CXR: Chest radiography
